# Risk factors for metabolic syndrome in individuals with recent-onset psychosis at disease onset and after 1-year follow-up

**DOI:** 10.1038/s41598-022-15479-x

**Published:** 2022-07-06

**Authors:** Yolanda Alonso, Carmen Miralles, M. José Algora, Alba Valiente-Pallejà, Vanessa Sánchez-Gistau, Gerard Muntané, Javier Labad, Elisabet Vilella, Lourdes Martorell

**Affiliations:** 1 Hospital Universitari Institut Pere Mata, Ctra. de l’Institut Pere Mata, s/n, 43206 Reus, Catalonia Spain; 2 Institut d’Investigació Sanitària Pere Virgili-CERCA, 43204 Reus, Catalonia Spain; 3 Universitat Rovira i Virgili (URV), 43201 Reus, Catalonia Spain; 4 Centro de Investigación Biomédica en Red en Salud Mental, CIBERSAM-Instituto de Salud Carlos III, 28029 Madrid, Spain; 5 Institut de Biologia Evolutiva, IBE, Universitat Pompeu Fabra, UPF, 08003 Barcelona, Catalonia Spain; 6 Hospital de Mataró, Consorci Sanitari del Maresme, Fundació Parc Taulí, 08340 Mataró, Catalonia Spain; 7 Institut de Innovació i Investigació Parc Taulí, I3PT, Translational Neuroscience Research Unit I3PT-INc-UAB, Institut de Neurociències, Universitat Autònoma de Barcelona, 08193 Barcelona, Spain

**Keywords:** Psychosis, Risk factors

## Abstract

Metabolic syndrome (MetS) is a cluster of parameters encompassing the most dangerous heart attack risk factors, associated with increased morbidity and mortality. It is highly prevalent in recent-onset psychosis (ROP) patients. In this pilot study, we evaluated MetS parameters (fasting glucose, high-density lipoprotein (HDL) cholesterol (HDL-c), fasting triglycerides, waist circumference, and systolic and diastolic blood pressure), clinical symptoms, pharmacological treatment, lifestyle, and inflammatory markers in 69 patients with ROP and 61 healthy controls (HCs). At baseline, waist circumference (p = 0.005) and fasting triglycerides (p = 0.007) were higher in patients with ROP than in HCs. At the 1-year follow-up, patients showed clinical improvement, with a reduction in the positive and negative syndrome scale (PANSS) score (p < 0.001), dietary intake (p = 0.001), and antipsychotic medication dose (p < 0.001); however, fasting glucose (p = 0.011), HDL-c (p = 0.013) and waist circumference worsened (p < 0.001). We identified sex, age, BMI, dietary intake, physical activity, daily tobacco use, daily cannabis use, and antipsychotic doses as risk factors contributing to baseline MetS parameters. After 1-year follow-up, those factors plus the PANSS and Calgary Depression Scale for Schizophrenia (CDSS) scores were associated with MetS parameters. Further studies are needed to understand the contributions of the studied risk factors in patients with ROP at onset and during disease progression.

## Introduction

Metabolic syndrome (MetS) is defined by the co-occurrence of several risk factors associated with increased morbidity and mortality, including high blood pressure, high blood glucose, excess fat around the waist, high triglycerides and low levels of high-density lipoprotein (HDL) cholesterol (HDL-c)^1^. The prevalence of MetS in young adults is approximately 5–7%, with low HDL-c being the most prevalent MetS component (41.2%), followed by elevated blood pressure (26.6%) and abdominal obesity (23.6%)^2^. An increased prevalence of MetS of 13.2% has been recently identified in antipsychotic-naïve patients with first-episode psychosis (FEP)^3^. Similarly, blood pressure, waist circumference and fasting glucose have been reported to be slightly higher in antipsychotic-naïve subjects with a high risk of psychosis than in the general population^4^, and low HDL-c has been reported in drug-naïve adolescents with FEP^5^. Finally, a study of the evolution of MetS risk factors in patients with FEP revealed a progressive worsening of the MetS profile, specifically in abdominal circumference and HDL-c and triglyceride levels, with the most notable changes in the first year^6^. Sex, the presence of affective symptoms, early disease onset, antipsychotic polypharmacy, and the use of antidepressants or mood stabilizers were also identified as possible risk factors for the worsening of the metabolic profile^6^.

In individuals with psychotic disorders, MetS has been associated with genetic variation^7,8^, mitochondrial dysfunction^9–12^, antipsychotic treatment^13–16^, clinical severity^6,17^, unhealthy lifestyle habits^18–20^ and a primary alteration of the innate immune system (imbalance between pro- and anti-inflammatory responses)^21,22^. Particularly, patients with FEP show an energy balance exceeding 26%^23^ and consumed more saturated fat and exercised less^24–26^. Poor dietary habits have been associated with increased BMI and abdominal obesity^24^ and cholesterol levels^26^. In addition, tobacco and alcohol consumption has been associated with some components of MetS in FEP patients^27,28^ and in the general population^29,30^. The relationship between cannabis use and the metabolic components of MetS is not well understood^24,31,32^. Finally, inflammation may also play a role in the pathogenesis of psychoses, with an elevation of specific cytokines and inflammatory mediators such as fibrinogen and C-reactive protein (CRP)^33–36^. The baseline levels of interleukin-6, CRP and leptin predicted incident MetS in schizophrenia patients, and it has been suggested that patients at increased risk of suffering the cardiometabolic adverse effects of antipsychotics could be identified before treatment initiation^36,37^.

Few prospective studies have investigated the relationships between lifestyle habits and metabolic parameters in the early stages of psychosis. One study reported a worsening of MetS components after 1 year of treatment, with antipsychotic treatment and low aerobic fitness identified as significant contributing risk factors^38^. Nonetheless, the progression of MetS parameters is of great interest in the context of psychotic disorders, and preventive strategies based on improving lifestyle might not be sufficient to prevent future cardiometabolic alterations^39^. Our study evaluated the progression of MetS parameters at the initial stages of psychosis in patients seen in an early intervention unit. Specifically, we aimed (1) to compare the MetS components between patients with recent-onset psychosis (ROP) at the beginning of the intervention and healthy controls (HCs); (2) to assess the evolution of MetS components in patients with ROP after a 1-year follow-up; and (3) to identify the contributions of demographic factors, lifestyle, clinical factors and antipsychotic medication to MetS components at the two time points.

## Results

### Differences in MetS parameters between patients with ROP and HCs at baseline

Patients and controls were age matched but differed in sex distribution, ethnic makeup, civil status and working status. Table 1 compares the baseline data of patients with ROP and HCs. We observed higher fasting triglycerides (*U* = 1525; p = 0.007) and waist circumference (*F* = 8.0; p = 0.005) in ROP patients and lower systolic blood pressure (*U* = 1474; p = 0.036). In the sex-stratified sample, we found significant differences in waist circumference (*t* =  − 2.0; p = 0.048) and systolic (*U* = 463; p = 0.002) and diastolic blood pressure (*U* = 556; p = 0.024) in the male group. The prevalences of MetS components in male patients were as follows: high triglycerides 17.3%, low HDL-c 13.5%, high systolic blood pressure 13.4%, high diastolic blood pressure 9.6%, high waist circumference 9.6% and high fasting glucose 1.9%. In female patients, the prevalence rates were as follows: high triglycerides 11.8%, low HDL-c 29.4%, and high waist circumference 47.0%. No women presented alterations in fasting glucose or systolic and diastolic blood pressure. In addition, BMI was higher in ROP patients (*U* = 1471; p = 0.025), but in the sex-stratified analysis, only females showed statistically significant differences between ROP and HC groups (*U* = 126; p = 0.018). At baseline, the overall prevalence of MetS was 2.9% and 4.9% in ROP patients and HCs, respectively.

**Table 1:** Demographic and clinical data of the study sample.

	ROP N = 69	HC N = 61	Statistics
**Sex, N (%)**			
Male	52 (75.4)	33 (54.1)	χ^2^ = 6.5; **p = 0.011**
Female	17 (24.6)	28 (45.9)	
Age (years), Mdn [IQR]	24.0 [9]	25.0 [8]	*U* = 1946; p = 0.557
**Ethnic group, N (%)**			
Caucasian	49 (71.0)	59 (96.7)	χ^2^ = 9.9; **p = 0.002**
Non-Caucasian	14 (20.3)	2 (3.3)	
**Civil status, N (%)**			
Single/separated	54 (85.7)	37 (60.7)	χ^2^ = 10.0; **p = 0.002**
Cohabiting/married	9 (14.3)	24 (39.3)	
**Working status, N (%)**			
Student/worker	21 (38.9)	54 (88.5)	χ^2^ = 31.1; **p < 0.001**
Unemployed	33 (61.1)	7 (11.5)	
**Daily substance use, N (%)**			
Tobacco	46 (66.7)	18 (29.5)	χ^2^ = 18.9; **p < 0.001**
Cannabis	24 (34.8)	4 (6.6)	χ^2^ = 15.7; **p < 0.001**
Alcohol	11 (15.9)	1 (1.6)	χ^2^ = 8.1; **p = 0.005**
BMI, (kg/m^2^), Mdn [IQR]	22.8 [4.0]	21.9 [3.5]	*U* = 1471; **p = 0.025**
**Physical activity**			
MET-min/week, Mdn [IQR]	1300 [1453]	2466 [2106]	*U* = 625; **p < 0.001**
**Dietary intake**			
Kcal/day, Mdn [IQR]	2304 [1060]	1723 [494]	*U* = 1077; **p < 0.001**
**MetS parameters**			
Fasting glucose (mg/dL), M ± SD	78.9 ± 8.8	78.9 ± 10.7	*t* = − 0.004; p = 0.996
HDL-c (mg/dL), M ± SD	57.9 ± 17.9	59.5 ± 14.5	F = 0.002; p = 0.968^1^
Fasting triglycerides (mg/dL), Mdn [IQR]	85.0 [68.0]	74.0 [36.0]	*U* = 1525; **p = 0.007**
Waist circumference (cm), M ± SD	83.1 ± 9.1	78.1 ± 8.9	F = 8.0; **p = 0.005**^1^
Systolic blood pressure (mmHg), Mdn [IQR]	110.0 [19]	115.0 [15]	*U* = 1474; **p = 0.036**
Diastolic blood pressure (mmHg), Mdn [IQR]	70.0 [15]	70.0 [10]	*U* = 1635; p = 0.195
MetS prevalence, *N (%)*	2 (2.9)	3 (4.9)	χ^2^ = 0.4; p = 0.665
**Inflammatory markers**			
CRP (mg/L), Mdn [IQR]	1.1 [1.9]	0.8 [1.8]	*U* = 1166; p = 0.487
Fibrinogen (mg/L), M ± SD	262.7 ± 64.1	272.4 ± 56.1	*t* = 0.8; p = 0.415

*ROP* recent-onset psychosis, *HC* healthy control, *N* number of cases; *M* mean; *SD* standard deviation, *Mdn* median, *IQR* interquartile range, *BMI* body mass index, *MET* metabolic equivalent task, *MetS* metabolic syndrome, *HDL-c* high-density lipoprotein cholesterol, *CRP* c-reactive protein.

Significant differences are indicated in boldface.

^1^Sex was considered in the comparison of groups.

### Lifestyle and inflammatory markers in patients with ROP and HCs at baseline

Patients showed higher calorie intake (*U* = 1077; p < 0.001) and lower physical activity levels (*U* = 625; p < 0.001). Greater proportions of ROP individuals showed daily consumption of tobacco (χ^2^ = 18.9; p < 0.001), cannabis (χ^2^ = 15.7; p < 0.001) and alcohol (χ^2^ = 8.1; p = 0.005). When the sample was stratified by sex, these differences were maintained for all variables in the two groups.

Differences in inflammatory markers (CRP and fibrinogen) between patients and the control group were not observed in either the whole sample or the sex-stratified analysis. Notably, CRP showed a partial correlation (controlling for sex) with triglyceride levels (r_partial_ = 0.237; p = 0.020) and waist circumference (r_partial_ = 0.266; p = 0.009), but fibrinogen was not correlated with any MetS component. The prevalence of patients with MetS increased from 3.2% at baseline to 7.9% after 1 year.

### MetS parameters at baseline based on pharmacological treatments

At baseline, 79.4% of the patients were treated with antipsychotics, 17.5% with antidepressants, 14.3% with mood stabilizers and 30.2% with benzodiazepines. The most common antipsychotic was risperidone (38.9%), the most common antidepressant was venlafaxine (33.3%), and the most common mood stabilizer was valproic acid (63.6%). The median [IQR] dose of antipsychotics in chlorpromazine equivalents (eCPZ) was 360 [206] mg/day. HDL-c was the only MetS parameter that differed between patients with neuroleptic treatment and treatment-naïve patients (*U* = 250; p = 0.024). No differences in MetS parameters were observed between patients with or without pharmacological treatment with antidepressants, benzodiazepines, or mood stabilizers. Inflammatory markers and lifestyle habits also did not differ between patients with and without neuroleptic treatment.

### Progression of MetS parameters at the 1-year follow-up of patients with ROP

Psychopathological evaluation with the positive and negative syndrome scale (PANSS) after 1 year of follow-up revealed that the positive PANSS score (PANSS-P) (*Z* =  − 4.148; p < 0.001), general PANSS score (PANSS-G) (*Z* =  − 3.171; p < 0.001), total PANSS score (PANSS-T) (*t* =  − 3.609; p < 0.001), and the State Trait Anxiety Inventory (STAI-S) (*t* = 2.670; p = 0.012) score significantly improved. Depressive symptoms of the CDS also improved, without reaching statistical significance (p = 0.326). These improvements in clinical symptoms were observed along with a decrease in the dose of antipsychotic medication after the 1-year follow-up (*Z* =  − 3.489; p < 0.001).

The comparison of MetS parameters between baseline and 1-year follow-up in patients with ROP is shown in Table 2. Patients exhibited a significant increase in fasting glucose (*t* = -2.624; p = 0.011) and waist circumference (*t* =  − 4.033; p < 0.001) and a decrease in HDL-c (*Z* =  − 2.471; p = 0.013). They also showed a significant increase in BMI (*t* =  − 7.597; p < 0.001). In the sex-stratified analysis, the differences in MetS components were significant only in men. The prevalence of MetS components in males at the 1-year follow-up was as follows: high fasting glucose 7.7%, high waist circumference 33.3%, low HDL-c levels 21.3%, high triglycerides 19.2%, high systolic blood pressure 7.7%, and high diastolic blood pressure 5.8%. The prevalence of MetS components in female patients was as follows: high waist circumference 64.7%, low HDL-c 17.6%, high triglycerides 11.8% and high diastolic pressure 5.8%. Women did not present alterations in fasting glucose or systolic blood pressure.

**Table 2:** Comparison of metabolic, lifestyle and clinical characteristics in patients with recent-onset psychosis (ROP) between baseline and the 1-year follow-up.

	Baseline	N	1-year follow-up	N	Statistics
**MetS parameters**					
Fasting glucose (mg/dL), M ± SD	78.7 ± 9.1	63	83.2 ± 11.0	*63*	*t* = − 2.624; **p = 0.011**
HDL-c (mg/dL), Mdn [IQR]	54.0 [21]	63	52.0 [16]	*63*	*Z* = − 2.471; **p = 0.013**
Fasting triglycerides (mg/dL), Mdn [IQR]	85.0 [62]	63	90 [71]	*63*	*Z* = − 1.698; p = 0.090
Waist circumference (cm), M ± SD	83.8 ± 9.1	63	88.3 ± 12.1	*63*	*t* = − 4.033; **p < 0.001**
Systolic blood pressure (mmHg), M ± SD	110.3 ± 12.9	63	111.2 ± 11.4	*63*	*t* = − 0.459; p = 0.648
Diastolic blood pressure (mmHg), M ± SD	68.0 ± 9.7	63	69.8 ± 10.0	*63*	*t* = − 1.299; p = 0.199
MetS prevalence, *N* (%)	2 (3.2)	63	5 (7.9)	63	χ^2^ = 0.605; p = 0.436
**Inflammatory markers**					
CRP (mg/L), Mdn [IQR]	1.1 [2.0]	24	2.6 [3.8]	*24*	*Z* = − 1.744; p = 0.081
Fibrinogen (mg/L)	254.1 ± 51	24	285.3 ± 70	*24*	*t* = − 1.854; p = 0.077
**Daily substance use, N (%)**					
Tobacco	40 (63.5)	63	36 (57.1)	63	χ^2^ = 0.298; p = 0.585
Cannabis	23 (36.5)	63	13 (20.6)	63	χ^2^ = 3.150; p = 0.076
Alcohol	11 (17.5)	63	4 (6.4)	63	χ^2^ = 2.724; p = 0.099
BMI, (kg/m^2^), M ± SD	23.4 ± 3.2	59	25.6 ± 3.9	59	t = − 7.597; **p < 0.001**
**Physical activity**					
MET-min/week, Mdn [IQR]	1386 [1666]	44	1040 [1584]	*31*	*Z* = − 0.625; p = 0.532
**Dietary intake**					
Kcal/day, M ± SD	2388 ± 729	56	1964 ± 719	*56*	*t* = 3.497; **p = 0.001**
**Psychopathology**					
PANSS-P, Mdn [IQR]	12.5 [10]	41	7.0 [2]	*41*	*Z* = − 4.148; **p < 0.001**
PANSS-N, Mdn [IQR]	17.0 [10]	41	13.0 [11]	*41*	*Z* = − 1.826; p = 0.068
PANSS-G, Mdn [IQR]	31.0 [15]	41	23.0 [13]	*41*	*Z* = − 3.171; **p = 0.002**
PANSS-T, Mdn [IQR]	60.5 [25]	41	46.5 [21]	*41*	*Z* = − 3.609; **p < 0.001**
CDSS, Mdn [IQR]	0.5 [5]	40	0.0 [1]	*40*	*Z* = − 0.982; p = 0.326
STAI-S	20.1 ± 10.9	30	15.7 ± 9.1	*30*	*t* = 2.670; **p = 0.012**
**Medication**					
Antipsychotics, N (%)	50 (79.4)	63	54 (85.7)	*63*	χ^2^ = 0.881; p = 0.348
eCPZ mg/day, Mdn [IQR]	360 [206]	63	150 [138]	*63*	*Z* = − 3.489; **p < 0.001**
Antidepressants, N (%)	11 (17.5)	63	7 (11.1)	63	χ^2^ = 1.037; p = 0.309
Benzodiazepines, N (%)	19 (30.2)	63	10 (15.9)	*63*	χ^2^ = 3.628; p = 0.057
Mood stabilizers, N (%)	9 (14.3)	63	11 (17.5)	63	χ^2^ = 0.238; p = 0.626

*M* mean, *SD* standard deviation, *Mdn* median, *IQR* interquartile range, *N* number of cases, *MetS* metabolic syndrome, *HDL-c* high-density lipoprotein cholesterol, *CRP* C-reactive protein, *BMI* body mass index, *MET* metabolic equivalent task, *PANNS-P* Positive and Negative Syndrome Scale, positive subscale, *PANSS-N* PANSS, negative subscale, *PANSS-G* PANSS, general subscale, *PANSS-T* PANSS total score, *CDSS* Calgary depression scale for schizophrenia, *STAI-S* state-trait anxiety inventory-state subscale, *eCPZ* equivalents of chlorpromazine doses.

Significant differences are indicated in boldface.

### Improvement of lifestyle habits in patients with ROP after 1 year of follow-up

Calorie intake improved after 1 year of follow-up (*t* = 3.497; p = 0.001) and, in the sex-stratified analysis, only in men (*t* = 3.215; p = 0.002); however, physical activity did not (Z =  − 0.625; p = 0.532). The number of individuals showing daily consumption of tobacco, cannabis and alcohol also improved, although the differences were not statistically significant. Patients had no differences between baseline and 1 year in the CRP or fibrinogen level, but their values increased after 1 year of follow-up. Similar to the findings at baseline, CRP 1 year later also showed a partial correlation with triglycerides (r_partial_ = 0.443; p = 0.039), waist circumference (r_partial_ = 0.574; p = 0.005), systolic pressure (r_partial_ = 0.533; p = 0.011) and diastolic pressure (r_partial_ = 0.516; p = 0.014), while fibrinogen was correlated with fasting glucose (r_partial_ = 0.467; p = 0.029).

### Risk factors contributing to MetS parameters at baseline

We conducted hierarchical multiple regression analysis with backward elimination to identify the best predictive model for the MetS components at baseline and after the 1-year follow-up. We included sex, age, BMI, calorie intake, physical activity, daily tobacco use, daily cannabis use, PANSS-T, CDSS score and eCPZ in the analysis as covariates. We did not include inflammatory markers because they did not show significant differences between patients with ROP and HCs. Table 3 shows the models at baseline. We obtained significant models for fasting glucose (AdjR^2^ = 0.299; p = 0.006), fasting triglycerides (AdjR^2^ = 0.269; p = 0.010), waist circumference (AdjR^2^ = 0.666; p < 0.001), systolic pressure (AdjR^2^ = 0.374; p = 0.003) and diastolic pressure (AdjR^2^ = 0.268; p = 0.026) and almost significant for HDL-c (AdjR^2^ = 0.106; p = 0.058). Age and eCPZ contributed to fasting glucose; tobacco contributed to triglyceride levels; BMI contributed to waist circumference; sex and diet intake contributed to systolic pressure; and finally, physical activity, tobacco use and cannabis use contributed to diastolic pressure.

**Table 3:** Linear model for predicting metabolic parameters in patients with ROP at baseline.

Risk factor	Fasting glucose			HDL-c			Fasting triglycerides			Waist circumference			Systolic pressure			Diastolic pressure		
	Adj.R^2^ = 0.299; F = 6.339; p = 0.006			Adj.R^2^ = 0.106; F = 3.976; p = 0.058			Adj.R^2^ = 0.269; F = 5.594; p = 0.010			Adj.R^2^ = 0.666; F = 50.913; p < 0.001			Adj.R^2^ = 0.374; F = 7.867; p = 0.003			Adj.R^2^ = 0.268; F = 3.806; p = 0.026		
	β	t	p	β	t	p	β	t	p	β	t	p	β	t	p	β	t	p
Sex													− 0.644	− 3.756	**0.001**			
Age	0.463	2.708	**0.013**															
BMI										0.824	7.135	** < 0.001**						
Dietary intake													− 0.386	− 2.253	**0.035**			
Physical activity																− 0.378	− 2.092	0.**049**
Tobacco							0.731	3.343	**0.003**							0.561	2.105	**0.048**
Cannabis							− 0.437	− 1.999	0.058							− 0.622	− 2.314	**0.031**
PANSS				− 0.377	− 1.994	0.058												
CDSS																		
eCPZ	0.482	2.817	**0.010**															

*ROP* recent-onset psychosis, *Physical activity* metabolic equivalents of task (MET)-min/week, *Dietary intake* Kcal/day, *Tobacco* daily consumption or not, *Cannabis* daily consumption or not, *PANSS* positive and negative syndrome scale, total score, *CDSS* Calgary depression scale for schizophrenia, *eCPZ* equivalent doses of chlorpromazine/day.

Significant p values are shown in boldface.

### Risk factors contributing to MetS parameters at the 1-year follow-up

After the 1-year follow-up, we conducted the same models to predict MetS components considering the risk factors at baseline (Table 4, panel A) and at the 1-year follow-up (Table 4, panel B) to elucidate their contribution to the model and their predictive potential. Significant findings were obtained for fasting glucose (AdjR^2^ = 0.436; p = 0.004), waist circumference (AdjR^2^ = 0.575; p = 0.001), systolic pressure (AdjR^2^ = 0.628; p < 0.001) and diastolic pressure (AdjR^2^ = 0.617; p < 0.001). Sex, age, PANSS-T and CDSS score contributed to fasting glucose; sex and PANSS-T contributed to waist circumference; sex, physical activity and tobacco and cannabis daily consumption contributed to systolic pressure; and finally, sex, age, and tobacco and cannabis daily consumption contributed to diastolic pressure. No significant models were obtained for HDL-c (AdjR^2^ = 0.117; p = 0.057) or triglyceride levels (AdjR^2^ = 0.120; p = 0.101).

**Table 4:** Linear models for predicting metabolic parameters in patients with ROP after a 1-year follow-up.

(A) Risk factor at baseline	Fasting glucose_1y			HDL cholesterol_1y			Fasting triglycerides_1y			Waist circumference_1y			Systolic pressure_1y			Diastolic pressure_1y		
	Adj.R^2^ = 0.436; F = 5.439; p = 0.004			Adj.R^2^ = 0.117; F = 4.044; p = 0.057			Adj.R^2^ = 0.120; F = 2.568; p = 0.101			Adj.R^2^ = 0.575; F = 6.187; p = 0.001			Adj.R^2^ = 0.628; F = 9.875; p < 0.001			Adj.R^2^ = 0.617; F = 7.767; p < 0.001		
	β	t	p	β	t	p	β	t	p	β	t	p	β	t	p	β	t	p
Sex	− 0.435	− 2.574	**0.019**							− 0.501	− 3.441	**0.003**	− 0.563	− 4.037	** < 0.001**	− 0.367	− 2.401	**0.029**
Age	0.622	3.137	**0.005**													0.554	3.639	**0.002**
BMI										0.282	2.016	0.060						
Dietary intake										− 0.294	− 1.972	0.065						
Physical activity													0.283	2.116	**0.049**	0.294	2.089	0.053
Tobacco							0.581	2.205	**0.039**				0.530	2.963	**0.009**	0.542	2.975	**0.009**
Cannabis							− 0.491	− 1.864	0.076	0.278	1.804	0.089	− 0.607	− 3.508	**0.003**	− 0.747	− 4.219	** < 0.001**
PANSS	0.606	3.471	**0.003**	− 0.394	− 2.011	0.057				0.534	3.651	**0.002**						
CDSS	− 0.418	− 2.159	**0.044**															
eCPZ										− 0.301	− 2.096	0.051						

(B) Risk Factor at 1-year follow-up	Fasting glucose_1y			HDL cholesterol_1y			Fasting triglycerides_1y			Waist circumference_1y			Systolic pressure_1y			Diastolic pressure_1y		
	Adj.R^2^ = 0.416; F = 4.320; p = 0.030			Adj.R^2^ = 0.849; F = 16.800; p < 0.001			Adj.R^2^ = 0.749; F = 11.445; p < 0.001			Adj.R^2^ = 0.909; F = 35.981; p < 0.001			Adj.R^2^ = 0.966; F = 50.237; p < 0.001			Adj.R^2^ = 0.769; F = 7.674; p = 0.013		
	β	t	p	β	t	p	β	t	p	β	t	p	β	t	p	β	t	p
Sex	− 0.481	− 1.938	0.079	0.762	4.723	**0.001**	− 0.744	− 4.439	**0.001**				− 0.501	− 6.136	**0.002**	− 0.492	− 2.295	0.062
Age_1y	0.730	2.853	**0.016**										0.630	5.865	0.**002**	0.558	2.967	**0.025**
BMI_1y							0.608	4.428	**0.001**	0.713	7.798	** < 0.001**				0.520	3.568	**0.012**
Dietary intake_1y							− 0.599	− 3.968	**0.003**							− 0.533	− 3.058	**0.022**
Physical activity_1y										0.264	2.898	**0.016**	0.444	4.871	**0.005**			
Tobacco_1y				− 0.535	− 4.126	**0.003**							− 0.413	− 6.198	**0.002**			
Cannabis_1y				0.426	2.983	**0.015**							0.464	5.301	**0.003**	0.426	2.356	0.057
PANSS_1y	0.552	2.224	**0.048**	− 0.372	− 2.881	**0.018**				0.224	2.673	**0.023**	0.568	4.770	**0.005**			
CDSS_1y				− 0.441	− 3.723	**0.005**	0.368	2.386	**0.038**	− 0.177	− 2.060	0.066						
eCPZ_1y													0.258	3.909	**0.011**	0.481	2.716	**0.035**

*ROP* recent-onset psychosis, *Physical activity* metabolic equivalents of task (MET)-min/week, *Dietary intake* Kcal/day, *Tobacco* daily consumption or not, *Cannabis* daily consumption or not; *PANSS* positive and negative syndrome scale, total score, *CDSS* Calgary depression scale for schizophrenia, *eCPZ* equivalent doses of chlorpromazine/day.

Significant p values are shown in boldface.

When considering the values of risk factors obtained after the 1-year follow-up, we observed better and more significant models for HDL-c (AdjR^2^ = 0.849; p < 0.001), fasting triglycerides (AdjR^2^ = 0.749; p < 0.001), waist circumference (AdjR^2^ = 0.909; p < 0.001), and systolic pressure (AdjR^2^ = 0.966; p < 0.001) but not for fasting glucose (AdjR^2^ = 0.416; p = 0.030) or diastolic pressure (AdjR^2^ = 0.769; p = 0.013), which showed better predictive values at baseline. Age and PANSS contributed to fasting glucose; sex, tobacco and cannabis daily consumption, PANSS-T and CDSS score contributed to HDL-c; sex, BMI, diet intake and CDSS score contributed to triglyceride levels; BMI, physical activity and PANSS-T contributed to waist circumference; sex, age, physical activity, tobacco and cannabis daily use, PANSS-T and eCPZ doses contributed to systolic pressure; and finally, age, BMI, diet intake and eCPZ contributed to diastolic pressure.

## Discussion

The increased rates of MetS recently reported in antipsychotic naïve FEP patients^3^ compared to those reported in HCs clearly suggest that there is an urgent need to investigate risk factors that contribute to MetS parameters that would allow preventive health strategies. With this aim, we investigated risk factors associated with MetS at the onset of psychotic disorders. Patients who participated in the study were treated in an early intervention unit, so the results obtained reflect the real-world situation in most developed countries in which early intervention programs (EIPs) are in place. At the beginning of the intervention, we identified two MetS parameters that were higher in ROP patients than HCs: fasting triglycerides and waist circumference. After the 1-year follow-up, HDL-c decreased, and fasting glucose and waist circumference increased, indicating that these three MetS parameters worsened as the psychotic illness progressed. It is worth mentioning that fasting glucose levels were similar between ROP patients and HCs at baseline but worsened in patients at the 1-year follow-up. Additionally, both systolic and diastolic blood pressure presented lower or equal values in ROP patients than in HCs and did not change after 1 year of patient follow-up. Our results are in accordance with previous studies that identified higher waist circumference and lipid abnormalities regardless of FEP treatment^40^. HDL-c has been highlighted by some authors as an early-onset indicator of cardiometabolic risk in drug-naïve patients^5^, and low HDL-c levels have also been described as the most prevalent MetS component in healthy young people and might be a target in the primary prevention of MetS^2^. In our study, waist circumference and both systolic and diastolic blood pressure were significantly worse in males, while no differences were observed in females. However, studies with larger samples found that male FEP patients showed higher systolic blood pressure and lower HDL-c, while female patients showed higher waist circumference and lower HDL-c levels^6^.

We did not find differences between ROP patients and HCs in the two inflammatory markers investigated, CRP and fibrinogen, though CRP showed significant correlations with triglyceride level and waist circumference. This finding suggests a possible relationship between the circulating inflammatory marker CRP and metabolic alterations related to MetS, as has been suggested in subjects exhibiting MetS in the general population^41^, and furthermore, CRP has been identified as a predictor of future sequelae of MetS, such as atherosclerotic cardiovascular disease^42^. CRP and fibrinogen increased at the 1-year follow-up, but not significantly. CRP has been suggested as a biomarker for onset risk, as well as a risk factor for cardiovascular disease, MetS and increased nicotine dependence in smokers with schizophrenia. For these reasons, the use of anti-inflammatory strategies in those patients showing increased CRP levels at baseline has been proposed^22^.

Concerning lifestyle, patients consumed more calories and exercised less, and more patients consumed tobacco, cannabis and alcohol on a daily basis than HC subjects. These results are in line with previously published studies and indicate the high prevalence of unhealthy habits in the early stages of psychotic disorders and the necessity of developing specific and intensive programs to improve health habits^24–26^. Some remarkable results of the present study were the changes at the 1-year follow-up in patients with ROP. After attending the early intervention unit for 1 year, patients significantly reduced their calorie intake. Additionally, the number of subjects consuming cannabis daily significantly decreased, as did the number of those consuming tobacco and alcohol, but not significantly. In addition, they showed a significant clinical improvement, as the scores of the clinical scales PANSS and STAI-S significantly decreased, and their antipsychotic doses were reduced. Conversely, fasting glucose, HDL-c and waist circumference were significantly worse. The patients’ BMI also increased. Of note, after one year of attending the early intervention unit, patients had not improved in the performance of physical exercise, and an intervention to improve this aspect could be beneficial to improve, or at least not worsen, the parameters of MetS. This aspect should be further investigated in future studies. Our results are difficult to compare with those of other studies for various reasons. A similar study conducted in patients with first-episode schizophrenia who also attended an outpatient clinic reported a significant increase in waist circumference and triglyceride levels after a 1-year follow-up, but the researchers did not observe significant changes in the other MetS parameters^38^. However, their patients exhibited improved dietary habits and positive and negative psychopathological symptoms but significantly increased antipsychotic medication use during this period, so their results are not comparable to ours. A rapid increase in body weight gain and waist circumference during the first year with stabilization afterward has been reported in patients with FEP in a 2-year follow-up study^6^. Some studies have suggested that sex is related to MetS development. Male rather than female FEP patients are more predisposed to insulin resistance and dyslipidemia^43^; MetS prevalence has been reported to be slightly higher in men than women younger than 50 years, and the trend reverses after 50 years of age^44^. Moreover, male sex has been related to the observed body weight gain and waist circumference increase in patients with FEP^6^. Additionally, men of white ethnicity appeared to have a particular vulnerability to the emergence of central obesity, exhibiting increased waist size^39^. In contrast, no differences in the prevalence of MetS between men and women at the 1-year follow-up have been reported, although more women fulfilled the waist circumference MetS criterion^38^. Along these lines, it is worth noting that the underrepresentation of women in clinical trials and the underutilization of paced therapy, for example, in women with ischemic heart disease, can greatly vitiate the interpretation of many epidemiological and clinical studies on sex and gender issues^45^. Our results show that waist circumference was the most prevalent MetS parameter in both men and women, although the increase was greater in men. Therefore, our study adds evidence that waist circumference measurement could be a suitable noninvasive parameter in the follow-up of metabolic risk in patients with FEP who attended mental health centers and the necessity of more effective psychoeducational programs to improve lifestyle habits.

We aimed to identify, by using regression analysis, the models that best explained the MetS parameters at baseline and after the 1-year follow-up. For this purpose, we used backward elimination regression to reduce the number of predictors while mitigating the multicollinearity problem. Notably, we identified different contributors to baseline MetS parameters. We found 5 of the 6 models to be statistically significant, with a mean of 1.8 risk factors contributing to each model. Thus, sex, age, BMI, dietary intake, physical activity, daily tobacco use, daily cannabis use, and eCPZ doses were risk factors that distinctly contributed to MetS parameters at baseline. After a 1-year follow-up, we created two different models for each MetS parameter, the first one using risk factor data obtained at baseline and the other with the data obtained at the 1-year follow-up. In the first situation, 4 out of 6 possible models were statistically significant, and the number of predictor risk factors averaged 3.8. In the second situation, all 6 models showed statistical significance, and the number of risk factors contributing to each model was 4.2. Notably, fasting glucose at 1 year was better predicted from baseline predictive factors.

This study has some limitations that must be addressed. First, the sample was small, and the sex distribution was unbalanced. Therefore, stratified analyses could not be properly performed. Additionally, the number of individuals included in the follow-up study along with the many predictors considered may classify this study as an exploratory study, and the results should be confirmed in larger samples. This study was conducted in patients with ROP, 92% of whom were on antipsychotic medication at the beginning of the study. Thus, even though it reflects a real-world situation, it may not reflect the situation in naïve patients. Moreover, a 1-year follow-up is short, and the results of educational interventions in lifestyle and their contribution to MetS parameters could need longer periods to be evaluated. Finally, other etiologic factors, such as genetic variants or personality traits, that might be implicated in the development of MetS abnormalities in patients with ROP were not studied. In this sense, it is worth mentioning a recent study that revealed, by using genome-wide association study (GWAS) data, a significant genetic correlation between MetS-related traits, CRP and schizophrenia^46^.

In conclusion, we found worse values of the MetS components triglycerides and waist circumference in patients with ROP than in HCs at baseline. The prevalence of abnormalities in MetS parameters in patients increased significantly during the first year of treatment despite improving lifestyle habits and decreasing antipsychotic medication. At 1 year, fasting glucose, waist circumference and HDL-c worsened. All the risk factors studied contributed to aggravated MetS parameters at the 1-year follow-up, although they made a modest contribution at baseline. The evidence shows that MetS components worsen from the early stages of psychotic disorders. It is necessary to investigate the contribution of risk factors for MetS if we are to develop and apply preventive programs for at-risk individuals.

## Methods

### Design

A cross-sectional study was conducted between patients with ROP (less than 3 months after being included in the EIP) and HCs. In addition, a longitudinal study was performed only in the patient group with two time points: baseline and after 1 year of follow-up.

### Participants

**Figure 1:**
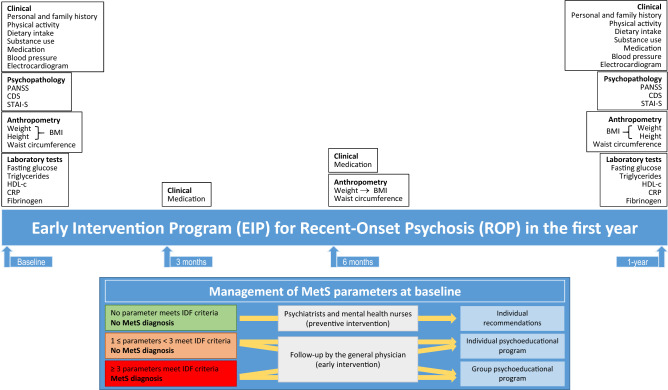
Characteristics and interventions of the early intervention program (EIP) in relation to metabolic syndrome (MetS) in the first year of follow-up of patients with recent-onset psychosis (ROP).

The study included 69 outpatients with ROP and 61 HCs. Patients aged 18 to 35 years were seen at the early psychosis program, outpatient clinic facility of the Hospital Universitari Institut Pere Mata, Reus, Catalonia, Spain, and were diagnosed using the DSM-IV criteria. Figure 1 shows the main characteristics and interventions of the EIP regarding MetS. The control group included 61 age-matched, unrelated HC individuals who were friends of the patients (90%) or were university students (10%). Table 1 shows the characteristics of the sample. Inclusion criteria for both patients and controls included the ability to speak Spanish or Catalan well enough to complete the assessment and no significant history of organic factors implicated in the etiology of psychotic symptoms. Patients were included if they had a DSM-IV diagnosis for psychotic disorders and less than 3 months of antipsychotic pharmacological treatment. HCs were included if they scored lower than 7 on the General Health Questionnaire (GHQ-28)^47^. Exclusion criteria for both patients and controls included intellectual disability, pregnancy, substance dependence, physical disability, and the presence of cardiovascular, endocrine or metabolic disease. The local ethics committee approved the study in accordance with the ethical standards of the current (2013) version of the Declaration of Helsinki. A complete description of the study was given to participants before their inclusion in the study, and they were included if they gave written informed consent. We evaluated the patients at baseline and after 1 year of follow-up, while HCs were evaluated only at baseline. Six patients did not complete the follow-up study.

### Clinical assessment

The diagnosis was confirmed in patients using the psychiatric interview OPCRIT checklist v.4.0., which generates DSM-IV diagnoses for psychotic disorders. HCs were also screened by a trained psychiatrist to rule out past or current history of psychiatric disorders. In patients, psychopathological data were gathered with PANSS^48^, STAI^49^ and CDSS^50^. Pharmacological data regarding antipsychotic, antidepressant, benzodiazepine, and mood stabilizer use were also collected. Daily antipsychotic doses were converted into eCPZ, and benzodiazepine doses were converted into diazepam equivalents.

### Lifestyle

Food intake was registered using a 24-h recall obtained via clinical interview. We calculated the daily calorie and nutrient intake with CESNID 1.0 software as previously described^51^. We also obtained information on the frequency, intensity, and duration of physical activity of all participants over the past 7 days by the International Physical Activity Questionnaire short version (IPAQ-SF)^52^. Tobacco, cannabis, and alcohol use was categorized as follows: no use, sometimes, regularly or daily.

### MetS criteria

According to the International Diabetes Federation (IDF), the diagnosis of MetS requires the presence of central obesity (waist circumference ≥ 94 cm in males and ≥ 80 cm in females) plus any two of the four following factors: (i) elevated triglycerides (≥ 150 mg/dL or specific treatment for this lipid abnormality); (ii) reduced HDL-c (< 40 mg/dL in males and < 50 mg/dL in females or specific treatment for this lipid abnormality); (iii) elevated blood pressure (systolic ≥ 130 mmHg or diastolic ≥ 85 mmHg or treatment for previously diagnosed hypertension; and (iv) elevated fasting plasma glucose (≥ 100 mg/dL or previously diagnosed type 2 diabetes)^1^.

### MetS parameters

Fasting glucose, triglycerides and HDL-c were measured by spectrophotometry as previously described^33^. Weight, height, and waist circumference were obtained by physical examination by a trained nurse, and body mass index (BMI) was calculated as weight (kg)/height^2^ (m). Systolic and diastolic blood pressure were taken at the upper right arm in a seated position.

### Inflammatory markers

Fasting specimens were obtained by antecubital needle venipuncture (8–10 a.m.); CRP and fibrinogen levels were measured on the day of blood sampling in the morning. High-sensitivity CRP levels were quantified by immunoturbidimetry (Menarini Diagnósticos, S.A., Badalona, Barcelona, Spain), and fibrinogen levels were quantified using the Clauss method with the Gernon Hemofibrin L Kit (RAL Técnica para el laboratorio, S.A., Sant Joan Despí, Barcelona, Spain).

### Data analysis

Data were processed using IBM SPSS Statistics, v.28.0 (IBM Corp., Armonk, NY). Normality of variables was checked by the one-sample Kolmogorov–Smirnov test. Categorical variables were compared between patients with ROP and HCs at baseline and, in the ROP group, between baseline and 1-year follow-up by the χ^2^-squared test. Continuous variables that were normally distributed were compared by Student’s *t* test or by the general linear model if sex needed to be considered. The Mann–Whitney U test was used to compare groups when the dependent variable was either ordinal or continuous, but not normally distributed. The paired-samples *t* test or the Wilcoxon signed-rank test was used to compare baseline and 1-year follow-up among patients based on the normality of variables. We used Spearman’s rank correlation test to measure the strength of the relationship between variable pairs or partial correlation when sex was considered a confounding factor. Finally, multiple linear regression models with backward elimination were applied to identify the risk factors (independent variables) contributing to MetS components both at baseline and after 1 year of follow-up. Data are presented as adjusted correlation coefficients between the independent variables (predictors) and dependent variables (MetS component) (95% CI; p ≤ 0.05).

## Data Availability

The data collected for this study can be provided upon reasonable request.
